# Adherence properties and adhesin-encoding genes detected in enteroaggregative *Escherichia coli* (EAEC)

**DOI:** 10.1128/spectrum.02070-25

**Published:** 2025-10-27

**Authors:** Guilherme F. R. de Souza, Gustavo B. Bueno, Daiany R. P. de Lira, Iranildo do A. Fernandes, Henrique Orsi, Beatrice D. V. L. Souza, Bruna M. Luiz, Luísa Pereira, Luís F. dos Santos, Waldir P. Elias, Rodrigo T. Hernandes

**Affiliations:** 1 Instituto de Biociências, Universidade Estadual Paulista (UNESP), Botucatu, São Paulo, Brazil; 2 Centro de Bacteriologia, Instituto Adolfo Lutz 89119, São Paulo, São Paulo, Brazil; 3 Laboratório de Bacteriologia, Instituto Butantan 196591, São Paulo, São Paulo, Brazil; Fernando Navarro-Garcia, Cinvestav-IPN, Mexico City, Mexico

**Keywords:** Enteroaggregative *Escherichia coli*, adherence, biofilm, chain-like adherence

## Abstract

Enteroaggregative *Escherichia coli* (EAEC) is an enteropathogen that harbors a set of adhesins responsible for mediating adherence to epithelial cells in a stacked brick-like arrangement termed aggregative adherence (AA) pattern and is one of the main pathogens isolated from diarrheal patients worldwide. In the present study, we characterized 140 EAEC isolates, obtained from stool samples of individuals with diarrhea in Brazil, based on their phylogeny, adherence properties, and presence of major pilin-encoding genes for aggregative adherence fimbriae (AAF) and aggregate-forming pili (AFP). The majority of the EAEC isolates were assigned into the phylogroups A (57.9%), B1 (18.6%), and D (17.1%), while the serotypes O86:H2 (7.9%), O153:H2 (6.4%), O168:H4 (4.3%), O175:H28 (4.3%), and O73:H18 (3.6%) were the most frequent. A total of 78.6% of the isolates produced the AA pattern on HeLa cells and/or on the glass coverslips, and 69.3% were classified as biofilm producers on polystyrene. Interestingly, three isolates (2.1%) produced chain-like adherence (CLA), which is characterized by the formation of bacterial cell chains on the surface of epithelial cells. Major pilin-encoding genes were detected with the following frequencies: *aggA* (37.8%), *agg3A* (14.3%), *agg4A* (13.6%), *agg5A* (13.6%), *aafA* (9.3%), and *afpA* (8.6%). Additionally, four isolates (2.8%) harbored *agg3A* and *agg5A* concomitantly. Of note, all EAEC isolates producing CLA harbored *agg4A*. Our data indicate that the EAEC isolates studied have originated from several *E. coli* backgrounds, leading to the emergence of a heterogeneous group of enteropathogen. Moreover, we highlighted a distinct EAEC subgroup that produces CLA on infected cells.

**IMPORTANCE:**

Enteroaggregative *Escherichia coli* (EAEC) is a major cause of both acute and persistent diarrhea worldwide, with a particularly high prevalence in developing countries. In this study, we performed a comprehensive molecular and phenotypic characterization of 140 EAEC isolates obtained from both pediatric and adult patients with diarrheal disease. Our findings support the knowledge that EAEC represents a heterogeneous group of isolates that have emerged from distinct *E. coli* genetic backgrounds, primarily within phylogroups A, B1, and D. Although most isolates exhibited the characteristic aggregative adherence (AA) pattern on infected epithelial cells, a subset displayed the chain-like adherence (CLA) phenotype and carried the *aatA*, *aggR*, and *agg4A* genes. This scenario highlights the need for continuous surveillance to monitor the emergence of novel virulence strategies employed by EAEC to adhere to host cells and cause disease.

## INTRODUCTION

Enteroaggregative *Escherichia coli* (EAEC) is one of the six pathotypes of diarrheagenic *E. coli* (DEC), comprising isolates that adhere to epithelial cells in the aggregative adherence (AA) pattern, which is characterized by the organization of bacterial cells in a stacked brick-like arrangement (1). The phenotypic assay currently used to detect the AA pattern is routinely performed using HeLa or HEp-2 cells, with a 3-hour incubation period for bacterial-epithelial cell interaction (2, 3). D-mannose is included in this assay to inhibit the adherence mediated by type 1 fimbriae (T1F), which are produced by most of the *E. coli* isolates (4). Numerous EAEC isolates can exhibit the AA pattern not only on the epithelial cell monolayers but also on the glass coverslip surfaces (5).

 EAEC has been recognized as an agent of endemic and traveler’s diarrhea and responsible for foodborne outbreaks of diarrhea (6–8). Also, growth impairment and intestinal inflammation have been associated with asymptomatic EAEC colonization in children of low-income countries (9).

 The characteristic AA pattern is mainly mediated by a family of chaperone-usher fimbriae termed aggregative adherence fimbriae (AAFs), which have been categorized to date into five distinct variants (AAF/I to AAF/V) (10–14). The genes encoding the proteins involved in the AAFs biogenesis, i.e., chaperone, usher, minor, and major pilin subunits, are located in a high molecular weight plasmid known in literature as the aggregative adherence plasmid or pAA (6, 11). While the genes encoding the proteins required for AAF/I, III, IV, and V biogenesis are organized in an operon (15), for AAF/II, these genes are separated into two distinct genomic regions of the pAA (16).

 Several adhesins have been identified in EAEC strains as key structures involved in the establishment of the aggregative adherence (AA) pattern. These include non-fimbrial adhesins, such as aggregative protein 58 (Ap58) and heat-resistant agglutinin 1 (Hra1), as well as fimbrial adhesins like T1F, *E. coli* common pilus (ECP), long polar fimbriae (LPF), YehD fimbriae (YDF), and the type IV pilus Pil (17–25). However, it is important to mention that these adhesins are not exclusive to EAEC, as they are also found in other *E. coli* pathotypes. In EAEC, they function as accessory adhesive structures that contribute to the AA pattern in cooperation with other adhesins, particularly the AAFs. Unlike accessory adhesins, AAFs are unique to EAEC and therefore are considered promising targets for diagnostic and preventive strategies (26, 27).

 Right after the description of AAF/I (10), a transcriptional regulator of the AraC family of DNA binding proteins, designated AggR, was identified and considered indispensable for AAF/I production in the EAEC 17-2 strain (28). Furthermore, subsequent studies have demonstrated that in addition to the pAA genes, AggR also regulates chromosomal virulence factor-encoding genes in the prototypical EAEC strain 042, such as the *aaiA*-Y operon, which encodes a putative type VI secretion system (20, 29, 30). Of importance, the *aggR* gene is also located in the pAA and has been currently used for subclassification of the EAEC isolates into typical and atypical, with the *aggR* gene present only in the former group of isolates (31).

 In addition to the AAFs-encoding genes and *aggR*, the pAA harbors several other virulence factor-encoding genes, such as *aap* (antiaggregation protein dispersin), *astA* (heat-stable enterotoxin EAST1), *pet* (plasmid-encoded toxin), *sepA* (*Shigella* extracellular protein), and the *aatABCDP* operon (type 1 secretion system apparatus responsible for dispersin secretion) (6).

 More recently, a type 4 fimbriae, termed aggregate-forming pili (AFP), was identified in a Shiga toxin–producing *E. coli* (STEC)/EAEC hybrid isolate of serotype O23:H8 and was associated with the establishment of the AA pattern on the surface of infected cultured epithelial cells (32). The proteins involved in AFP biogenesis are encoded by a set of 15 genes located on the pAFP plasmid and organized in a single operon. In addition to the *afp* operon, this plasmid also harbors the *afpR* gene that encodes an AraC-like regulator protein required for AFP production (32). To date, genes from the *afp* operon and/or *afpR* have only been detected in atypical EAEC isolates (33–35).

 Numerous eukaryotic host cell surface structures have been identified as potential binding sites for the various bacterial adhesins described to date. Notable examples include glycoproteins, glycolipids, and several components of the extracellular matrix, such as laminin, fibronectin, and collagen (36–38). In the context of EAEC, one study demonstrated that AAF/II binds to fibronectin in a dose-dependent manner (39). However, the specific eukaryotic receptors for other AAF variants, as well as for AFP, have yet to be identified and remain to be investigated.

 Because not all EAEC strains are virulent in volunteer studies (40) and virulence factors described in prototypical EAEC strains are found in variable frequencies among clinical strains, defining truly virulent EAEC strains remains a challenge (6, 11, 33, 41–44). Therefore, it is important to characterize circulating EAEC strains of different settings to define relevant genetic markers for diagnosis and prevention strategies. Hence, in the present study, we aimed to characterize a collection of 140 EAEC isolates, obtained from stool samples of individuals with diarrhea in Brazil, focusing on the determination of the O:H serotype, assignment into the distinct *E. coli* phylogroups, and occurrence of several adhesin-coding genes, as well as their ability to adhere to HeLa cells and produce biofilm on the abiotic surface.

## MATERIALS AND METHODS

### EAEC isolates

 A total of 140 EAEC isolates were obtained from stool samples of patients with acute diarrhea during the Food and Water Borne Diseases Laboratory’s epidemiological surveillance activities conducted at the Adolfo Lutz Institute (IAL), São Paulo, Brazil, from 2017 to 2023. These isolates were routinely selected in the laboratory using a multiplex PCR to detect DEC pathotypes (45). Those isolates harboring the *aatA* gene were classified as EAEC and stored at −80°C in lysogeny broth (LB) containing 30% glycerol. Further, they were classified as typical or atypical due to the presence and absence of the *aggR* gene, respectively. The presence of *aggR* was investigated by PCR DNA amplification using the primers, conditions, and controls described in Table 1. The reactions were performed using 7.5 µL of GoTaq Green Master Mix (Promega, Madison, USA), 5.0 µL of nuclease-free water, 1 µL of template DNA, and 0.34 µM of each primer. Template DNA consisted of one bacterial colony grown on LB agar, resuspended in 200 µL of nuclease-free H_2_O, and boiled for 10 min. Amplified products were analyzed by 1% agarose gel electrophoresis stained with SYBR Safe DNA Gel Stain (Invitrogen/Thermo Fisher Scientific, CA, USA) and visualized using the inGenius LHR gel documentation imaging system (Syngene, UK).

**TABLE 1:** Primers and PCR conditions for gene amplification in the EAEC isolates^*a*^

Target genes	Primer sequence (5′ → 3′)	PCR conditions:		Amplicon size (bp)	Reference
		Annealing Temperature (°C)	Extension Time (s)		
*aatA*	CTGGCGAAAGACTGTATCAT	55	30	630	(46)
	CAATGTATAGAAATCCGCTGTT				
*aggR*	CAGCGATACATTAAGACG	52	30	326	This study (GenBank accession number: FN554766.1)
	CATCTGCAATAATAGCTAGAG				
*aggA*	CTTTGGGTTTAGTTAGTCTTCTATCTGG	53	45	241	This study (GenBank accession number: NZ_JACEFV000000000.1)
	ACCTGTTCCCCATAACCAGACC				
*aafA*	CAAGTGGAGCCGCTATTAATGC	56	30	401	This study (GenBank accession number: FN554766.1)
	TCCTGGTCGTAGTGGCATAG				
*agg3A*	GTATCATTGCGAGTCTGGTATTCAG	55	45	462	(12)
	GGGCTGTTATAGAGTAACTTCCAG				
*agg4A*	TCCATTATGTCAGGCTGCAA	55	45	411	(13)
	GGCGTTAACGTCTGATTTCC				
*agg5A*	CATGTTCATTATCTATTAGTTCGCC	55	45	215	(14)
	TCCACCGTACGTCGTCATTA				
*afpA*	TTTCAGAAGGGCTTGTCATTAATCG	55	30	144	(44)
	GACGATACTCATGACTTCGGAGA				
*afpR*	GTGAAGAACATTATTGAAGGGGGC	55	30	307	(32)
	CATCACTTAATCGCCAGCGTT				
*cseA*	CGCAAATGCCGCAACTGTA	55	30	348	(34)
	GCGTCTGGCAAATTCCAAC				

^*a*^ The following strains were used as positive controls: EAEC 042 for *aatA*, *aggR,* and *aafA *(11); EAEC 17-2 for *aggA *(10); EAEC RNT785 for *agg3A *(47); EAEC BA1116 for *agg4A *(34); EAEC BA120 for *agg5A *(34); UPEC-46 for *afpA* and *afpR *(35); and EAEC BA249 for *cseA *(34). *E. coli* HB101 was used as negative control in all reactions.

### EAEC serotyping

Somatic (O) and flagellar (H) antigens of the EAEC isolates were determined at the Adolfo Lutz Institute by serum agglutination using specific rabbit antisera (O1 to O188 and H1 to H56), as previously described (48). All antisera were produced at Adolfo Lutz Institute.

### Assignment of EAEC isolates into phylogroups

The 140 EAEC isolates were assigned into one of the distinct *E. coli* phylogroups (A, B1, B2, C, D, E, and F) using the quadruplex PCR detecting *chuA*, *yjaA*, TspE4.C2, and *arpA*, as previously described (49). Further, EAEC isolates classified into phylogroup B2 lacking the *yjaA* gene, or into phylogroup F, were submitted to an additional PCR to investigate the possibility of these EAEC isolates being reclassified into phylogroup G (50). Amplicons were analyzed and visualized as described for *aggR* detection.

### Detection of adhesin-encoding genes

 The EAEC isolates were tested for the presence of the following adhesin-encoding genes: *aggA*, *aafA*, *agg3A*, *agg4A*, *agg5A*, *afpA*, *cseA*, and *afpR*. The presence of these genes was investigated by PCR DNA amplification using specific primers and amplification conditions as described in Table 1. Amplicons were analyzed and visualized as described for *aggR* detection.

### Adherence assay

The adherence patterns of the 140 EAEC isolates on HeLa cells were determined using the methodology described by Cravioto (2), with modifications. Epithelial cells were cultured in 5% CO_2_ atmosphere, at 37°C, in DMEM (Dulbecco’s Modified Eagle Medium–high glucose, Sigma-Aldrich, MO, USA) containing 10% fetal bovine serum (FBS) (Sigma-Aldrich, MO, USA) supplemented with 1% PenStrep (10,000 U penicillin and 10 mg streptomycin/mL) (Sigma-Aldrich, MO, USA). For the adherence assays, 1 mL of HeLa cell suspension at a concentration of 1 × 10^5^ cells/mL was cultured for 48 h in a 24-well microplate containing glass coverslips, using the same medium. After the incubation period, the cells were washed six times with phosphate-buffered solution (PBS), followed by the addition of 1 mL of DMEM supplemented with 2% D-mannose (Sigma-Aldrich, MO, USA) and 2% FBS, and then infected with approximately 1 × 10^7^ CFU/mL of the bacterial cultures, previously grown in LB at 37°C for approximately 18 h. The adherence assay was performed in 5% CO_2_, at 37°C, for 3 h. After the incubation period, the wells were washed six times with PBS and fixed with methanol for 18 h, followed by staining with May-Grünwald (Sigma-Aldrich, MO, USA) and Giemsa (Dinâmica, SP, Brazil). The preparations were analyzed at 1,000× magnification by light microscopy with immersion oil.

The adherence patterns observed were classified as: aggregative adherence (AA), when bacteria adhered to the cells and coverslip in a stacked brick arrangement; coverslip aggregative adherence (AAcs), characterized by bacteria preferentially adhered to the coverslip in aggregative pattern; diffuse adherence (DA), when bacteria diffusely adhered to the cell surface; chain-like adherence (CLA), which is characterized by the formation of bacterial cell chains on the surface of epithelial cells; cell detachment (CD), when cells detached from the coverslip; and undefined adherence (UND), when bacteria sporadically adhered only to HeLa cells, not producing a well-established adherence pattern.

### Biofilm production

The biofilm production assay was performed according to the protocol described by Oliveira-Garcia (51), with modifications. The bacterial isolates were grown in LB at 37°C for approximately 24 h. Then, 10 µL of these cultures were incubated in a 96-well plate containing 200 µL of DMEM high-glucose (Cultilab Materiais Cultura Celular, Brazil) with 2% D-mannose and incubated at 37°C for 24 h. After this incubation period, the plates were washed once with PBS to remove non-adherent bacterial cells and subsequently fixed with 3% formaldehyde for 1 h. Afterward, the preparations were washed once with distilled water, dried, and stained for 20 min with 0.1% crystal violet (Sigma-Aldrich, MO, USA). Further, the preparations were washed twice with distilled water and resuspended in methanol for 10 min. The optical density was measured at the wavelength of 570 nm (OD_570_) in an ELISA microplate reader (Biotek/Agilent Technologies, CA, USA). The isolates were classified according to Yang (52), in which the optical density cutoff (ODc) was defined as three times the standard deviation plus the mean optical density (OD) of the *E. coli* HB101, used as negative control. Based on the ODc obtained, the isolates were then classified as non-producer (≤ ODc), weak (> ODc ≤ 2 x ODc), moderate (> 2 x ODc ≤ 4 x ODc), strong (> 4 x ODc ≤ 8 x ODc), very strong (> 8 x ODc ≤16 x ODc), and extremely strong (> 16 x ODc) biofilm producer, respectively.

### Statistical analysis

The association between typical and atypical EAEC and AA production was assessed using Fisher’s exact test (two-tailed). Moreover, the association between distinct adherence patterns or levels of biofilm formation and the presence of specific AAF variants or AFP was assessed using the test for difference in proportions with continuity correction.

All statistical tests used to evaluate the association between distinct genetic profiles and phenotypic features were performed using the software SAS version 9.4 (SAS Institute Inc., NC, USA) and R version 4.0.2 (R Core Team, https://www.r-project.org), with *P* < 0.05 considered statistically significant.

## RESULTS

**Fig 1:**
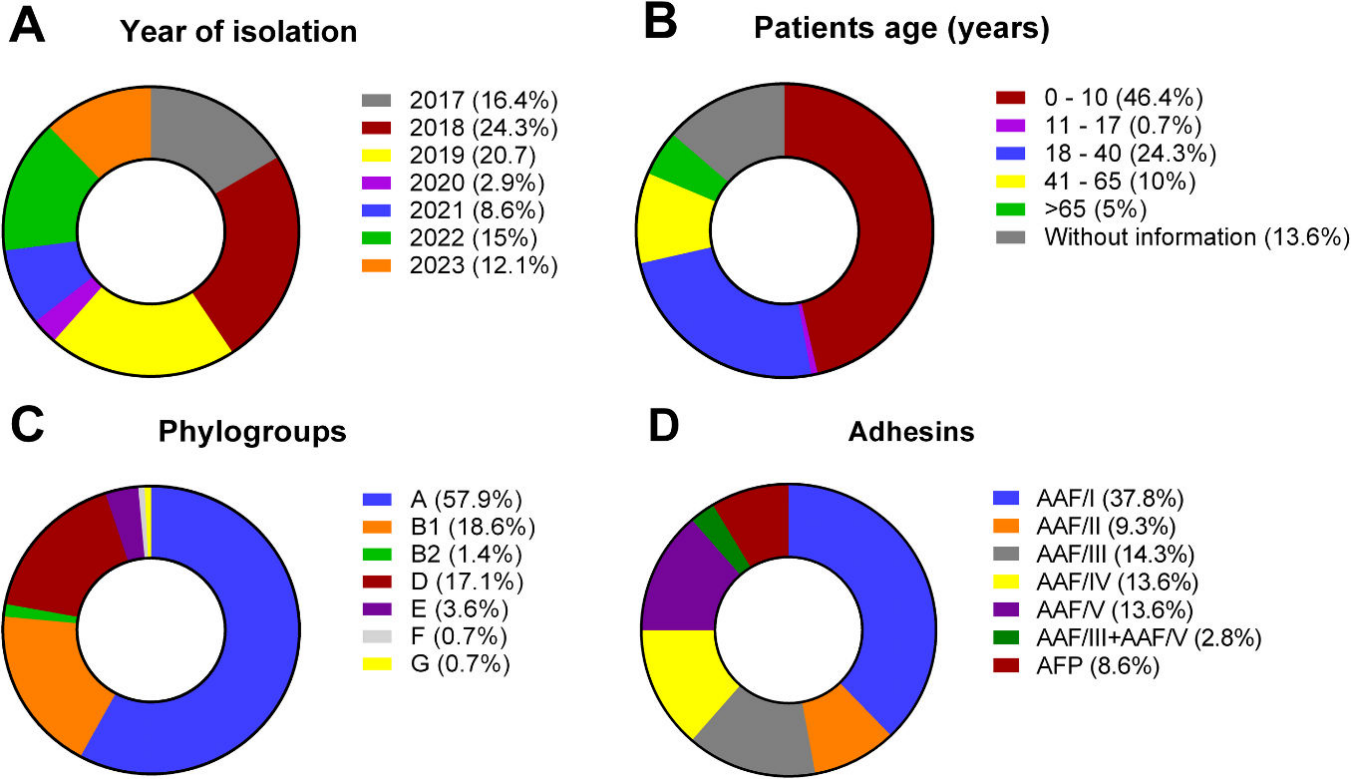
Demographic data (**A and B**) and molecular characteristics (**C and D**) of the EAEC isolates studied. Note that the majority of the Brazilian EAEC isolates were obtained from children up to 10 years old (46.4%), assigned into the phylogroups A (57.9%), B1 (18.6%), and D (17.1%) and harbored the AAF/I adhesin (37.8%).

 In the present study, we analyzed a total of 140 EAEC isolates obtained between 2017 and 2023 from stool samples of Brazilian patients with acute diarrhea, including children and adults, with most of these subjects being children up to 10 years of age (46.4%) (Fig. 1A and B). Other bacterial enteropathogens, such as *Salmonella* spp., *Shigella* spp., *Campylobacter* spp., *Aeromonas* spp., and *Plesiomonas* spp., were not detected in any of the stool samples collected in this study.

The vast majority of EAEC isolates harbored the *aggR* gene (91.4%) and were then classified as typical EAEC, while only 8.6% were classified as atypical (Table 2). A total of 89 EAEC isolates (63.6%) belonged to 28 distinct serogroups, in addition to 31 (22.1%) O-non-typeable (ONT) and 20 (14.3%) rough (OR) isolates (Table S1). The most frequent serogroups detected were O168 (10.0%), O86 (7.9%), O153 (6.4%), O92 (4.3%), O175 (4.3%), and O73 (3.6%). Considering the somatic and flagellar antigens completely identified, the most frequent serotypes observed among the EAEC isolates studied were O86:H2 (7.9%), O153:H2 (6.4%), O168:H4 (4.3%), O175:H28 (4.3%), and O73:H18 (3.6%) (Table S1).

**TABLE 2:** Identification of adhesin-encoding genes in EAEC isolates from distinct *E. coli* phylogroups and serotypes

Genes investigated	Phylogroups (No. of EAEC)	Serotypes^*a*^ (No. of EAEC)	No. (%) of EAEC
**EAEC isolates harboring** ***aggR*** **(*****n*** **= 128)**			
*aggA*	A (45)	O153:H2 (8), O168:HNM (8), OR:HNM (8), O168:H4 (6), O20:H30 (2), O21:H2 (2), O78:H2 (2), O92:H33 (2), O92:HNM (2), O3:H2 (1), O73:H18 (1), O78:HNM (1), ONT:H2 (1), ONT:HNM (1)	53 (37.8%)
	B1 (1)	ONT:H10 (1)	
	D (4)	O15:H6 (3), O15:H2 (1)	
	E (1)	O130:H27 (1)	
	F (1)	O153:H2 (1)	
	G (1)	OR:HNM (1)	
*aafA*	B1 (13)	O175:H28 (6), O181:H28 (2), ONT:HNT (2), O181:H16 (1), ONT:H25 (1), OR:HNM (1)	13 (9.3%)
*agg3A*	A (7)	ONT:HR (4), O92:H33 (2), O176:HNM (1)	20 (14.3%)
	B1 (3)	O104:H4 (3)	
	D (7)	O86:H2 (5), ONT:H18 (1), OR:H6 (1)	
	E (3)	O99:H6 (3)	
*agg4A*	B1 (5)	O55:H21 (1), O59:H19 (1), O173:H31 (1), ONT:H19 (1), OR:H19 (1)	19 (13.6%)
	D (13)	OR:H18 (5), O73:H18 (4), ONT:H18 (2), O44:H18 (1), O106:H18 (1)	
	E (1)	OR:H2 (1)	
*agg5A*	A (13)	O86:H2 (6), ONT:H10 (5), O3:HNM (1), ONT:H2 (1)	19 (13.6%)
	B1 (4)	ONT:H21 (3), O64:HNM (1)	
	B2 (2)	O25:H4 (2)	
*agg3A + agg5A*	A (4)	ONT:HNM (4)	4 (2.8%)
**EAEC isolates devoid of** ***aggR*** **(*****n*** **= 12)**			
*afpA + afpR*	A (12)	O4:H16 (1), O55:H25 (1), O103:H43 (1), O117:H32 (1), O145:H4 (1), O151:H11 (1), ONT:H10 (1), ONT:H30 (1), ONT:H32 (1), ONT:H33 (1), OR:H32 (1), OR:HNM (1)	12 (8.6%)

^*a*^ ONT, O-non-typeable; OR, rough (autoagglutinable) EAEC isolates; and HNM, non-motile EAEC isolates.

 Regarding the assignment of the EAEC isolates in distinct *E. coli* phylogroups, they were classified into phylogroups A (57.9%), B1 (18.6%), and D (17.1%). Less frequently, EAEC isolates from the phylogroups E (3.6%), B2 (1.4%), F (0.7%), and G (0.7%) were also observed (Fig. 1C).

 All typical EAEC isolates harbored at least one of the five distinct variants of the AAF pilin-encoding genes described so far, as follows: *aggA* (37.8%), *agg3A* (14.3%), *agg4A* (13.6%), *agg5A* (13.6%), and *aafA* (9.3%). Moreover, four EAEC isolates (2.8%) harboring the *agg3A* and *agg5A* genes concomitantly were also detected (Fig. 1D), whereas the *cseA* gene, associated with the colonization factor CS22, was not detected in any of the isolates analyzed in this study. On the other hand, all atypical EAEC isolates harbored the *afpA* and *afpR* genes, associated with AFP biogenesis.

 Despite the diversity of phylogroups and serotypes identified in the present study, some interesting associations between serotypes, phylogroup, and adhesin-encoding genes can be highlighted. In general, EAEC isolates from the same serotype harbored the same variant of the gene encoding the AAF pilin, as observed in EAEC isolates from the serotypes O153:H2 (nine isolates) and O168:H4 (six isolates) that harbored the *aggA* gene, as well as in EAEC isolates from the serotype O175:H28 (six isolates) that harbored *aafA* (Table 2). An interesting situation was observed in EAEC isolates from the serotype O86:H2 that were assigned into the phylogroups A (six isolates) and D (five isolates) and harbored the AAF pilin-encoding genes *agg5A* and *agg3A*, respectively (Table 2). A similar situation was observed with EAEC isolates of serotype O73:H18 (five isolates), where isolates from phylogroup D (four isolates) harbored the *agg4A* gene, and the only isolate assigned to the phylogroup A harbored *aggA*. Another point that deserves to be highlighted is the EAEC isolates of serotype O92:H33 (four isolates), classified into phylogroup A, in which the occurrence of isolates harboring *aggA* (two isolates) and *agg3A* (two isolates) was observed, as shown in Table 2.

**Fig 2:**
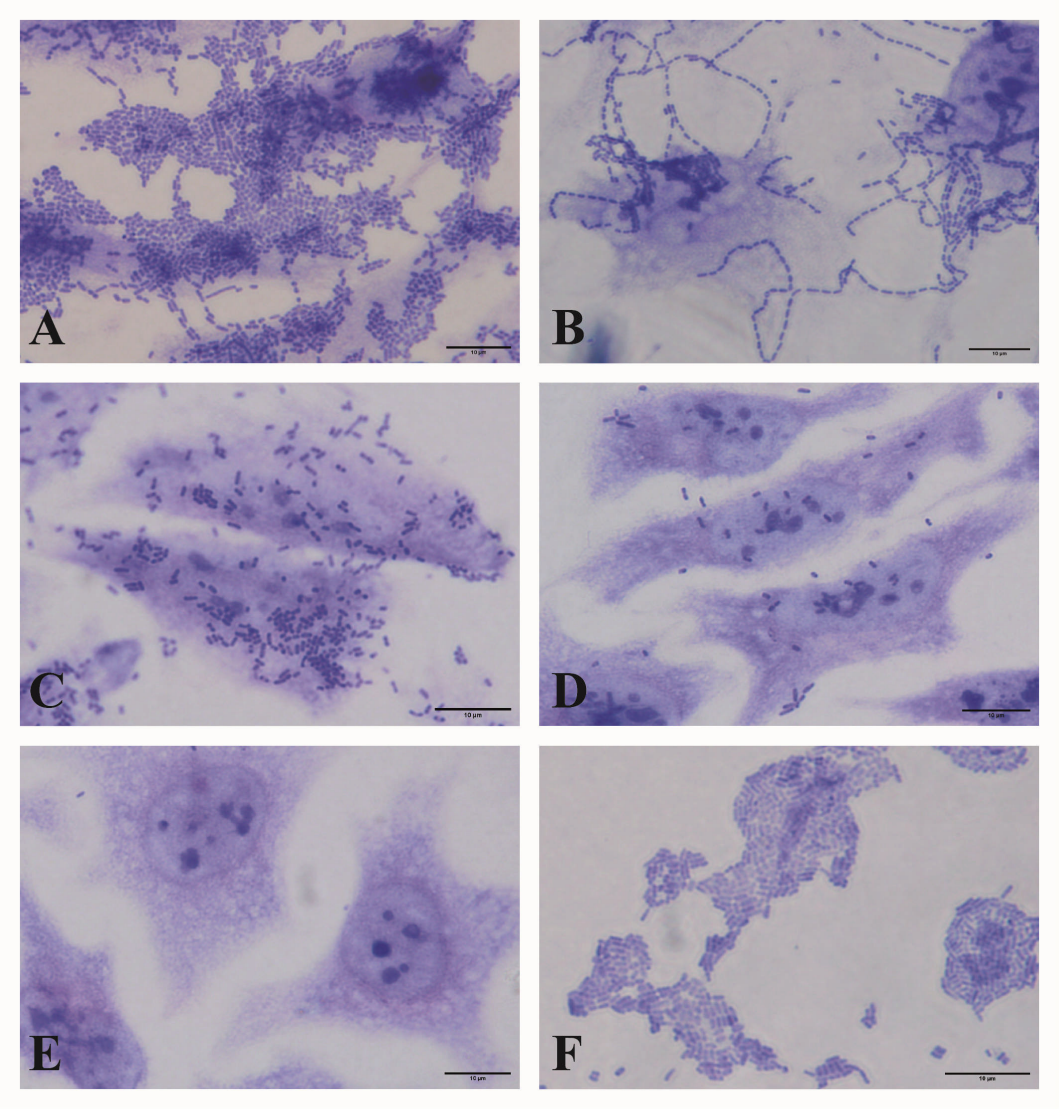
Distinct patterns of adherence displayed by the EAEC isolates in adherence assays performed with HeLa cells. The adherence patterns are represented as follows: (**A**) aggregative adherence (AA); (**B**) chain-like adherence (CLA); (**C**) diffuse adherence (DA); (**D**) undefined adherence (UND); (**E**) non-adherent (NA); and (**F**) EAEC that promoted cell detachment and produced the aggregative adherence pattern on glass coverslips surface (CD + AAcs). Scale bar = 10 µm.

The majority of EAEC isolates tested exhibited the AA pattern on HeLa cells (70.7%). Less frequently, isolates displaying CLA (2.1%) or DA (0.7%) patterns were identified. Eleven isolates (7.9%) promoted cell detachment (CD) and adhered exclusively to the glass coverslip surface, producing the AA pattern in this context, with this pattern being referred to in the present study as CD+AAcs. Additionally, 12 isolates (8.6%) demonstrated sporadic adherence to HeLa cells and were classified as undetermined (UND). Non-adherent isolates (9.3%) and those associated with cell detachment (0.7%) were also observed (Table 3; Fig. 2). Of importance, the AA pattern was significantly more frequent in typical EAEC isolates compared to atypical ones (82.8% vs 33.3%; *P* = 0.0005).

**TABLE 3:** Adherence patterns on HeLa cells and biofilm formation among the EAEC isolates studied

Adherence pattern/biofilm formation	Adhesin-encoding genes							Total (*n* = 140)	*P*-value^*a*^
	EAEC isolates harboring *aggR* (*n* = 128)						EAEC isolates devoid of *aggR*		
	AAF/I *aggA* (*n* = 53)	AAF/II *aafA* (*n* = 13)	AAF/III *agg3A* (*n* = 20)	AAF/IV *agg4A* (*n* = 19)	AAF/V *agg5A* (*n* = 19)	AAF/III + AAF/V *agg3A* + *agg5A* (*n* = 4)	AFP *afpA* (*n* = 12)		
Adherence pattern^*b*^									
AA	38 (71.7%)^1^	12 (92.3%)^1^	15 (75%)^1^	8 (42.1%)^1^	18 (94.7%)^1^	4 (100%)^1^	4 (33.3%)^2^	99 (70.7%)	*P* = 0.0001
CLA	0^1^	0^1^	0^1^	3 (15.8%)^2^	0^1^	0^1^	0^1^	3 (2.1%)	*P* = 0.003
DA	0	0	0	1 (5.3%)	0	0	0	1 (0.7%)	*P* = 0.3784
CD + AAcs	10 (18.9%)^1^	0^2^	1 (5%) (1)	0^2^	0^2^	0^2^	0^2^	11 (7.9%)	*P* = 0.0217
UND	3 (5.7%)	1 (7.7%)	3 (15%)	2 (10.5%)	1 (5.3%)	0	2 (16.7%)	12 (8.6%)	*P* = 0.7505
NA	1 (1.9%)^1^	0^1^	1 (5%)^1^	5 (26.3%)^2^	0^1^	0^1^	6 (50%)^2^	13 (9.3%)	*P* < 0.0001
CD	1 (1.9%)	0	0	0	0	0	0	1 (0.7%)	*P* = 0.9847
Biofilm formation									
Non-biofilm producer	15 (28.3%)^1^	0 (0.0%)^1^	7 (35%)^1^	8 (42.1%)^1^	2 (10.5%)^1^	0 (0.0%)^1^	11 (91.7%)^2^	43 (30.7%)	*P* < 0.0001
Weak biofilm producer	13 (24.5%)^1/2^	1 (7.7%)^2^	7 (35%)^1/2^	6 (31.6%)^1/2^	8 (42.1%)^1/2^	3 (75%)^1^	1 (8.3%)^2^	39 (27.9%)	*P* = 0.0480
Moderate biofilm producer	24 (45.3%)^1^	5 (38.5%)^1^	5 (25%)^1^	5 (26.3%)^1^	4 (21.1%)^1^	1 (25%)^1^	0 (0.0%)^2^	44 (31.4%)	*P* = 0.0166
Strong biofilm producer	1 (1.9%)^1^	7 (53.8%)^2^	1 (5%)^1^	0 (0.0%)^1^	5 (26.3%)^2^	0 (0.0%)^1^	0 (0.0%)^1^	14 (10%)	*P* < 0.0001

^*a*^ Percentages (%) followed by the same superscript number (1 and/or 2) do not differ at the 5% level by the proportion difference test with continuity correction.

^*b*^ Distinct adherence patterns displayed on HeLa cells: AA, aggregative adherence; CLA, chain-like adherence; DA, diffuse adherence; UND, undefined adherence; NA, non-adherent; CD, cell detachment; and CD + AAcs, EAEC that promoted cell detachment and produced the aggregative adherence pattern only on glass coverslips.

Considering the occurrence of the genes encoding the pilin subunits of the five distinct AAF types, the AA pattern was more frequently observed among EAEC isolates harboring the *agg5A* (94.7%), *aafA* (92.3%), and *agg3A* (75%) genes. Additionally, only EAEC isolates harboring *agg4A* produced the CLA pattern on HeLa cells. We also observed four isolates harboring *agg3A* and *agg5A* concomitantly, with all of them producing the AA pattern (Table 3).

**Fig 3:**
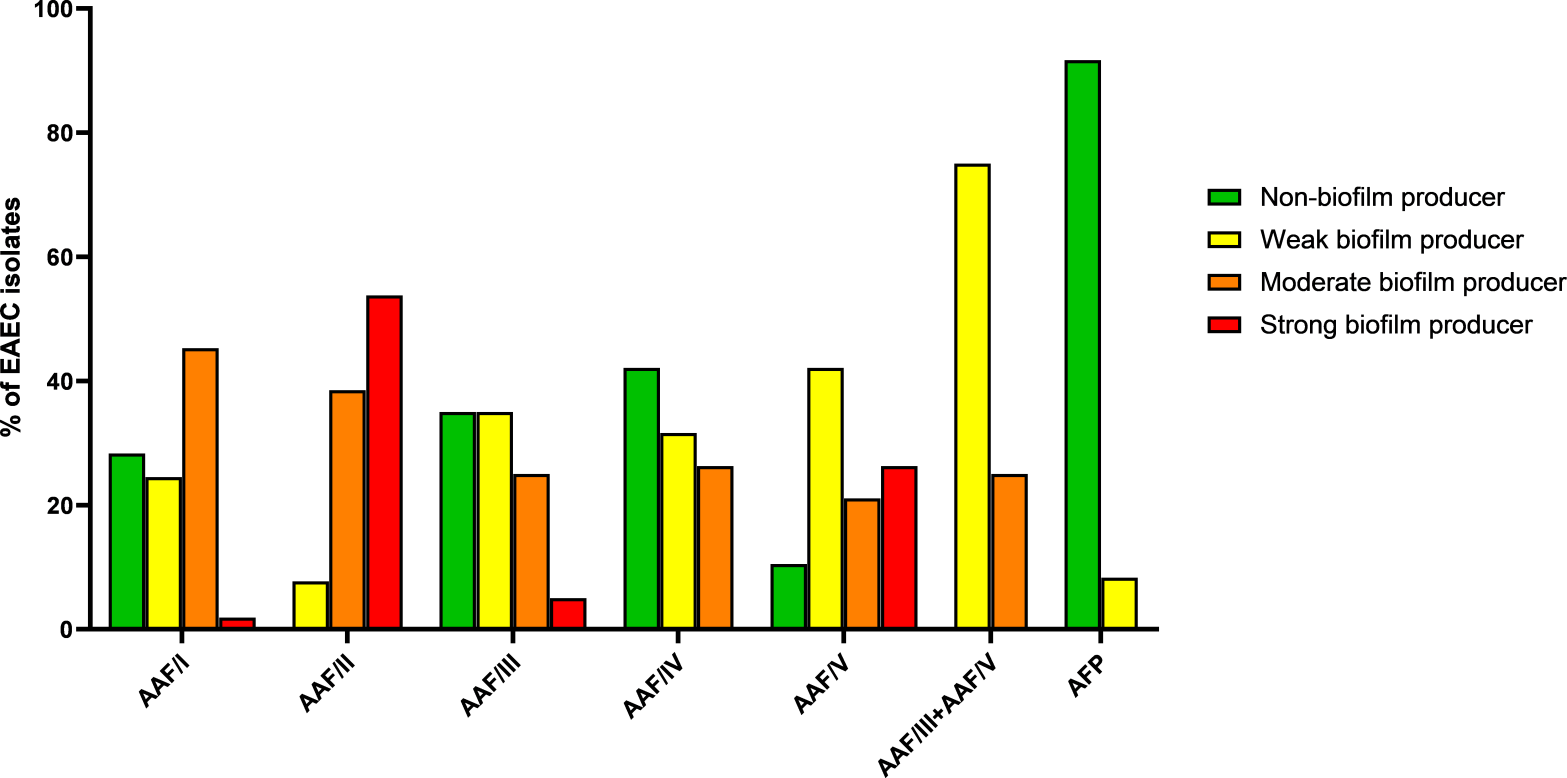
Biofilm production by the EAEC isolates studied. EAEC isolates carrying AAF/II were those that presented the highest frequency of strong biofilm producers, followed by EAEC isolates carrying AAF/V and AAF/III, while the highest frequency of non-biofilm producers was observed among those harboring AFP.

Regarding the biofilm production on abiotic surface, the isolates were classified as follows: moderate (31.4%), weak (27.9%), and strong (10%) biofilm producers, in addition to the occurrence of EAEC isolates that did not produce biofilm under the conditions tested (30.7%) (Table 3). A higher percentage of strong biofilm producers was observed among EAEC isolates harboring AAF/II (53.8%) and AAF/V (26.3%) compared to those producing other EAEC-associated fimbriae (*P* < 0.0001). The vast majority of EAEC isolates that harbored the *agg3A* and *agg5A* genes concomitantly were classified as weak biofilm producers (75%), and most of the EAEC isolates harboring AFP were unable to produce biofilm (91.7%; *P* < 0.0001) under the conditions tested in this study (Table 3 and Fig. 3).

## DISCUSSION

EAEC is one of the main DEC pathotypes isolated from patients with both acute and persistent diarrhea worldwide (33, 45, 53–57), with a particularly high prevalence in developing countries (6, 42). A limitation of most previous epidemiological studies, both in Brazil (33, 54, 58, 59) and in other countries (55), is that they predominantly focused on characterizing EAEC isolates from children under 10 years of age. However, a Brazilian study published in 2019 (45) demonstrated that EAEC can also be isolated from adults with diarrhea and may represent a significant etiological agent in this age group. In line with this evidence, our study included both children and adults with diarrhea and found that more than 40% of the EAEC isolates obtained from stool samples came from adult patients (6, 33, 45, 53–57).

The definition of this pathogen is based on two central points: (i) the production of the AA pattern on HeLa or HEp-2 infected cells and/or (ii) the detection of molecular markers present in the pAA, mainly the *aatA* and/or *aggR* genes (1, 26). As already observed in other studies (34, 60, 61), our data clearly reflect that these two methods do not present an absolute agreement.

Despite this, the difficulty in performing bacterial adherence assays to identify the AA pattern production on epithelial cells has resulted in the increasing use of molecular methods for the diagnosis of EAEC (62, 63). An interesting scenario resulting from this reality is the identification of isolates that fit the molecular definition of typical EAEC (*aatA*^+^/*aggR*^+^) but produce CLA on cultured epithelial cells. This may represent an important subgroup within this pathotype, as these isolates have been obtained from individuals with diarrhea (34, 61, 64). It is important to mention that in our study, the AA pattern was more frequently detected among typical than atypical EAEC isolates, similar to what has been observed by others (34, 61).

One important aspect to be considered regarding EAEC is the fact that most of its virulence factors are encoded by genes present in a high molecular weight plasmid designated pAA (6, 11). Our data showing the EAEC isolates classified into several *E. coli* phylogroups and serotypes clearly indicate that the pAA has been acquired countless times by distinct *E. coli* backgrounds, thus forming a very heterogeneous group of bacteria. A concerning aspect to be considered is the pAA acquisition by other DEC pathotypes, thereby originating hybrid isolates such as STEC/EAEC and EPEC/EAEC (65–69), which, due to the combination of virulence factors, could culminate in the emergence of potentially more pathogenic bacteria. No less important is the pAA acquisition by extraintestinal pathogenic *E. coli* (ExPEC), originating hybrid ExPEC/EAEC isolates (70), which have been increasingly isolated in the last decade from cases of cystitis and pyelonephritis (71–75), as well as bloodstream infection (27, 76–78). Collectively, these data suggest that *E. coli* isolates with EAEC markers are not restricted to the gastrointestinal tract but, instead, have emerged as an important agent of extraintestinal infections.

The discovery of five distinct AAF variants (AAF/I–AAF/V) during the last two decades has almost completely elucidated the repertoire of adhesin involved in the AA pattern production on HeLa and/or HEp-2 cells by typical EAEC isolates (34, 43, 61). The prevalence of these variants may differ significantly among the different EAEC collections studied, depending on the period in which the study was conducted and the geographic region from which these EAEC isolates were obtained (26, 34, 43, 61, 79, 80). An important point to consider is the difference observed in the frequency of the *aggA* gene (which encodes the AAF/I major pilin) in the present study (37.9%) compared to a previous study from our laboratory that detected this gene in 2.7% of the strains (61). Of note, several published studies have pointed to the existence of AAF/I subvariants (26, 81, 82). Based on this observation, we hypothesize that the primers used in our previous study (61) did not allow the amplification of all AAF/I subvariants, unlike those used in the present study, which were designed with this perspective in mind. Therefore, we believe that the low frequency of *aggA* observed in our previous study (61) does not reflect the real frequency of AAF/I among Brazilian EAEC isolates as shown in other studies (47, 79, 83, 84). Although the *cseA* gene, which is part of a cluster encoding the colonization factor CS22, has been proposed as a genetic marker for defining EAEC along with *aggR* (26), it was not detected in any of the isolates studied.

On the other hand, only recently have the adherence strategies used by atypical EAEC isolates (*aatA*^+^/*aggR*^-^) begun to be unraveled, with the discovery of a type IV fimbriae, termed AFP, which is responsible for mediating the establishment of the AA pattern on infected epithelial cells (32, 35). In agreement with previous studies from the literature (32, 34, 35, 61), we also showed that genes responsible for encoding proteins involved in AFP biogenesis were exclusively detected among atypical EAEC isolates. This observation certainly reinforces the use of genes from the *afp* operon (mainly *afpA* and *afpR* genes) as a genetic marker to be considered for the diagnosis of atypical EAEC in the routine of a clinical microbiology laboratory.

As previously reported by our group (61), only typical EAEC isolates harboring the *agg4A* gene produced CLA in adherence assays performed with HeLa cells. However, whether AAF/IV participates in the establishment of the CLA pattern is a question that remains to be elucidated. Currently, our laboratory is sequencing a collection of CLA-producing EAEC isolates to gain deeper insights into their genomic architecture, to investigate their phylogenetic relation with other EAEC isolates, and to identify the genetic determinants responsible for the establishment of this distinct adherence phenotype.

Although EAEC strains present only one AAF type, a study evaluating a collection of 162 Danish EAEC identified isolates harboring the major pilin-encoding genes for AAF/III (*agg3A*) and AAF/V (*agg5A*) concomitantly, both colocalized in the same pAA (85). Further, similarly to the findings in our study, EAEC *agg3A*^+^/*agg5A*^+^ isolates were also identified in other studies (34, 43, 61). However, whether both AAF pilins are present on the surface of these EAEC isolates, and in what arrangement, are questions that remain to be answered.

Biofilm production is an important attribute in EAEC pathogenesis, associated with strong intestinal colonization and persistence (86–88), and the AAF fimbriae are involved in this phenotype (43, 89, 90). Most EAEC isolates in this study showed the ability to form biofilm, but there was no absolute association between the AAF variant and the biofilm formation profile (strong, moderate, or weak biofilm forming). Similar results were described in other studies (43), where EAEC isolates with the same AAF type showed differential biofilm formation. Interestingly, in our study, most of the isolates harboring AFP were not capable of forming biofilm. Schüroff et al. (35) showed that an AFP-producing strain did not form biofilms on either plastic or glass surfaces when grown in different culture media, including urine. AFP has a role in the establishment of the AA pattern on epithelial cells (32, 35, 91), but apparently, its role in biofilm formation seems to be not necessary.

Here, we provide a comprehensive characterization of EAEC isolates circulating in Brazil, showing that these isolates may have originated from distinct *E. coli* backgrounds—mainly *E. coli* from the phylogroups A, B1, and D—comprising a highly heterogeneous group of bacteria. Furthermore, the establishment of the AA pattern in EAEC isolates carrying the *aatA* gene fully corresponds with the presence of one of the five AAF variants or AFP. Of importance, we identified a subgroup of EAEC isolates harboring the *agg4A* gene and producing CLA on infected HeLa cells, although the role of AAF/IV in the establishment of this phenotype has not yet been investigated.

## Data Availability

The authors confirm that the data supporting the findings of this study are available within the article and its supplementary materials. Moreover, the raw data used to generate graphs and tables are available in the Butantan Institute Repository at the following link: https://repositorio.butantan.gov.br/handle/butantan/13560

## SUPPLEMENTAL MATERIAL

Table S1 spectrum.02070-25-s0001.docx Classification of the EAEC isolates studied into the distinct serotypes.

ASM does not own the copyrights to Supplemental Material that may be linked to, or accessed through, an article. The authors have granted ASM a non-exclusive, world-wide license to publish the Supplemental Material files. Please contact the corresponding author directly for reuse.
