# Supplementary material for: Adherence properties and adhesin-encoding genes detected in enteroaggregative Escherichia coli (EAEC)
Source: Microbiol Spectr. 2025 Oct 27;13(12):e02070-25. doi: 10.1128/spectrum.02070-25 (PMC12671079; doi:10.1128/spectrum.02070-25)
Supplement: Table S1 — Classification of the EAEC isolates studied into the distinct serotypes. [file spectrum.02070-25-s0001.docx]

**SUPPLEMENTARY MATERIAL**

**Table S1.** Classification of the EAEC isolates studied into the distinct serotypes

| **Serogroup**  **(No. of isolates)** | **H type** | **No. of isolates (%)** |
| --- | --- | --- |
| O3 (2) | H2 | 1 (0.7%) |
|  | HNM | 1 (0.7%) |
| O4 (1) | H16 | 1 (0.7%) |
| O15 (4) | H2 | 1 (0.7%) |
|  | H6 | 3 (2.1%) |
| O20 (2) | H30 | 2 (1.4%) |
| O21 (2) | H2 | 2 (1.4%) |
| O25 (2) | H4 | 2 (1.4%) |
| O44 (1) | H18 | 1 (0.7%) |
| O55 (2) | H21 | 1 (0.7%) |
|  | H25 | 1 (0.7%) |
| O59 (1) | H19 | 1 (0.7%) |
| O64 (1) | HNM | 1 (0.7%) |
| O73 (5) | H18 | 5 (3.6%) |
| O78 (3) | H2 | 2 (1.4%) |
|  | HNM | 1 (0.7%) |
| O86 (11) | H2 | 11 (7.9%) |
| O92 (6) | H33 | 4 (2.9%) |
|  | HNM | 2 (1.4%) |
| O99 (3) | H6 | 3 (2.1%) |
| O103 (1) | H43 | 1 (0.7%) |
| O104 (3) | H4 | 3 (2.1%) |
| O106 (1) | H18 | 1 (0.7%) |
| O117 (1) | H32 | 1 (0.7%) |
| O130 (1) | H27 | 1 (0.7%) |
| O145 (1) | H4 | 1 (0.7%) |
| O151 (1) | H11 | 1 (0.7%) |
| O153 (9) | H2 | 9 (6.4%) |
| O168 (14) | H4 | 6 (4.3%) |
|  | HNM | 8 (5.7%) |
| O173 (1) | H31 | 1 (0.7%) |
| O175 (6) | H28 | 6 (4.3%) |
| O176 (1) | HNM | 1 (0.7%) |
| O181 (3) | H16 | 1 (0.7%) |
|  | H28 | 2 (1.4%) |

Table S1. *Continued*

| **Serogroup**  **(No. of isolates)** | **H type** | **No. of isolates (%)** |
| --- | --- | --- |
| ONT (31) | H2 | 2 (1.4%) |
|  | H10 | 7 (5.0%) |
|  | H18 | 3 (2.1%) |
|  | H19 | 1 (0.7%) |
|  | H21 | 3 (2.1%) |
|  | H25 | 1 (0.7%) |
|  | H30 | 1 (0.7%) |
|  | H32 | 1 (0.7%) |
|  | H33 | 1 (0.7%) |
|  | HNM | 11 (7.9%) |
| OR (20) | H2 | 1 (0.7%) |
|  | H6 | 1 (0.7%) |
|  | H18 | 5 (3.6%) |
|  | H19 | 1 (0.7%) |
|  | H32 | 1 (0.7%) |
|  | HNM | 11 (7.9%) |
